# The Limits of the Optical Capacity of the Microscope

**Published:** 1877-11

**Authors:** Lester Curtis

**Affiliations:** Chicago.


### ARTICLE IV.

### *The Limits of the Optical Capacity of the Microscope.*

BY LESTER CURTIS, M. D., CHICAGO.

Two papers, one written by Professor Abbe, of Jena, and the other by the well known Professor Helmholtz, on the limits, beyond which, from the nature of light, it is impossible to carry the magnifying power of the microscope, with any profit, are at present attracting considerable attention among those interested in the use of the instrument. It has been suggested to me that a condensed statement of the results arrived at by these investigators might be of interest to those readers of the *Medical Journal and Examiner* who might not have access to the original papers.

The first difficulty that stands in the way of a very great amplification of an object, is the difficulty of obtaining sufficient illumination to render it clearly visible. The opening through which the object is viewed in a microscope diminishes just in proportion to the magnifying power. The size of this apparent aperture has nothing to do with the size of any of the glasses of the microscope, and only varies with the changes in the magnifying power of the instrument; it makes no difference in what way this magnifying power is obtained, whether by the use of higher power objectives, or higher eye-pieces, or by lengthening the tube of the instrument. The only circumstance that modifies the size of this orifice is what is called the angular aperture of the objective, an objective of a small angle having a smaller field than an objective of a larger angle. The size of this orifice can be seen by arranging the microscope as for an ordinary observation, and then removing the eye a short distance from the eye-piece and looking down it. One who has never tried it before will be surprised at the exceeding minuteness of the orifice through which the object seems to be seen. As long as this orifice is no smaller than the pupil of the eye, the image seen in the microscope suffers no loss of brightness by its increased magnification; when however, the orifice through which the object is viewed becomes smaller than the pupil, the image seen loses in brightness.

Supposing the diameter of the pupil to be about 1-12 of an inch, and the objective to have an aperture of 180 degrees, the amplification, to be equal to the pupil, would be 166.7.

The brightness of the image as the magnifying power increases, diminishes in the following ratio:

| | | | |
|---|---|---|---|
| For an amplification of | 166.7 | brightness | 1. |
| " " " " | 333.3, | " | 1 4 |
| " " " " | 500.0, | " | 1-9. |
| " " " " | 666.7, | " | 1-16. |

It will be easily seen that for the very great magnifying powers sometimes used there must be a great diminution of the brightness of the image, notwithstanding the appliances that are used to increase the light; and finally there must be a limit beyond which the amplification cannot go, from the fact that the image will be so dim as to prevent its structure being made out.

Along with this decrease in the size of the beam of light admitted to the eye is another difficulty, and that is the production of shadows within the eye itself. It is well known that if a narrow beam of bright light is admitted to the eye in a direction somewhat oblique to its axis, that the shadows of the retinal vessels can be seen. Now, in the case of an observer who is examining an object highly magnified, a narrow beam of light passes into the eye, and the shadows of the retinal vessels thus produced, and appearing as a vail over the object, must, as a matter of course, impair the clearness with which it is seen.

There is still another difficulty connected with this same diminution of the orifice through which a highly magnified object is seen, and that is diffraction. We are all aware that if we look across the edge of a screen at any brightly illuminated object there appears a narrow shadowy fringe along the edge of the screen. This fringe is called diffraction, and is always found where light passes by the border of any object. If we place two edges so closely together that these diffraction fringes overlap, and look at any bright object through the opening, we shall observe a great impairment in the clearness with which it is seen. A convenient experiment is to make a small hole in a card, which is thick enough to be quite opaque; or to scratch a fine line on a piece of glass which has been covered with some black varnish, or covered with turpentine and smoked; and then to look through the hole at a printed page, upon which a strong light has been thrown; it will be seen that the letters which were to be seen with the greatest ease before can now be scarcely read at all. The smallest aperture through which an object can be seen, without an overlapping of these diffraction fringes, and a consequent impairment of the image, is about one-twelth of an inch. As we diminish the orifice, the details of the object become more indistinct. Now this diffraction occurs around the edge of the orifice, through which we look at images in a microscope, in exactly the same manner that it does around the edges of the orifice in the card, through which we looked at the letters of our book, and when we come to the higher magnifying powers the orifice becomes exceedingly small. Supposing that the angular aperture of the glass is 180 degrees, the orifice through which we look when the object is magnified 1,000 diameters is 1-50th of an inch, for 5,000 diameters it is 1-250th of an inch. The impairment in the clearness of an image, seen through such holes, can easily be learned by trying the experiment of looking at the flame of a lamp through a hole of that size.

Another and more serious difficulty yet remains to be considered, and that is the modification which the light undergoes in passing through the object before it reaches the microscope. If the details of an object examined with the microscope are not less than 1-750th of an inch apart, the image of the object is seen in exactly the same way in a microscope that one would be in a telescope, and the image seen can be relied upon as being an accurate representation of the object; but, if these structures approach closer than this distance, a certain part of the light suffers a change by diffraction, just as the light passing through the small hole suffers diffraction. When the details of the structure approach to within the 1-2500 of an inch of each other, all the light passing through the object becomes diffracted. In this case its direction is changed, and in place of passing directly through the object, the light is spread out into a fan shape, as it would be by a series of minute prisms.

If now we focus the microscope upon such an object as, for instance, a series of fine lines ruled on glass, and then take out the eye-piece and look down the tube of the instrument, we shall see in the center of the object-glass an image of the object. If the lines are no finer than 1-750 of an inch apart, we shall see nothing else than a true image of the object, but as the lines grow finer we shall begin to see colored spectral images on each side of the true image, one color after another appearing as the lines grow finer, and when the lines reach a fineness of 1-2500 of an inch we shall have a complete series spectral images extending to the right and left of the true image, beginning with the violet nearest to the true image and extending to the red furtherest away. The distance of these spectral images from each other and from the true image varies inversely, as the fineness of the lines; if the lines are not very fine the colored spectral images will be closer together, and the finer the lines the further apart will be the spectral images. If now we take out the eye-piece and put a diaphragm down the tube of the microscope, just behind the object-glass, which cuts off a portion of these spectral images, and replacing the eye piece examine the object, we shall begin to lose in the clearness of the appearance of the image. If we put in a series of diaphragms which cut off one after another of the spectral images, some of the details disappear with each one of the spectral images which is cut off, and when the diaphragm is small enough to cut off all the spectral images, all the details disappear and the structure appears a blank. It is necessary, therefore, in order to make out any of the details of an object that at least one spectral image shall enter the object-glass with the true image, and, if the object-glass has not angular aperture enough to take in these two images, no structure can be seen, however great the magnifying power that may be employed. Value of angular aperture is not therefore, that it allows great obliquity of light to be used, which causes light and shadow, and so shows surface markings, but because it enables the objective to take in these spectral images, which are caused by the most intimate structure of the object. Another conclusion from this is, that when the details of an object are so fine as to cause a dispersion too great to be taken in by any object-glass, the limit of possible vision is reached. This limit for an objective of 180 degrees of angular aperture, used with light whose direction is very oblique to the axis of the instrument, is equal to one-half the length of a wave of light. With the use of the ordinary mixed white daylight, this limit would be 1-92000 of an inch. By the use of blue light alone, this distance might be reduced to 1-122000 of an inch, which is the size of the smallest object that can, under any circumstances, be seen by a microscope theoretically perfect.

There are some other points that may be brought out by the use of these diaphragms. By cutting off some of the spectral images, intermediate between the true image and the outer spectral images, the lines can be apparently doubled and quadrupled in number. In objects with lines crossing each other at various inclinations, many different appearances, all apparently clearly defined, can be produced by cutting off some of the spectral images by diaphragms of various kinds.

According to these investigators therefore, we have already reached the limit of the magnifying power of the microscope; no microscope can ever be made which will give a useful magnifying power greater than some now in use; indeed Professor Abbe thinks that a magnifying power of seven or eight hundred diameters is all that can ever be used with profit.

Nobert's lines, ruled upon glass at a distance of 1-112000 of an inch apart, have been seen, and that is as near the theoretical limit as we can ever expect to attain.

Finally, according to these investigations, we must revise the opinions we have had in regard to the details of the structure of many minute microscopic objects, and confess that there is no certainty that the image of them which we see in the microscope represents their structure truthfully.

It may be well to mention, however, that in one or two well authenticated instances, objects appear to have been seen finer than the limits considered possible by this theory.—*Chicago Med. Jour. and Examiner.*

